# A cautionary note for claims about the microbiome’s impact on the “self”

**DOI:** 10.1371/journal.pbio.2006654

**Published:** 2018-09-12

**Authors:** Emily C. Parke, Brett Calcott, Maureen A. O’Malley

**Affiliations:** 1 Philosophy, School of Humanities, University of Auckland, Auckland, New Zealand; 2 Department of Philosophy, Charles Perkins Centre, University of Sydney, Sydney, Australia; 3 LaBRI, University of Bordeaux, Bordeaux, France; 4 History and Philosophy of Science, Carslaw Building, University of Sydney, Sydney, Australia

## A cautionary note for claims about the microbiome’s impact on the “self”

In their essay, “How the microbiome challenges our concept of self,” Rees and colleagues [1] argue that recent discoveries about the microbiome have far-reaching effects on our understanding of self and what it means to be human. They claim these effects are so profound that they require a new mode of operation for the arts and humanities: “a breakdown of the anachronistic barriers between the natural and the human sciences.” While we strongly support interdisciplinary collaboration between the arts and sciences, the authors overinflate the microbiome’s influence in ways that are conceptually and empirically problematic.

The empirical problems come from statements about the microbiome’s causal power as a unitary entity, which we think need to be more carefully phrased. The conceptual problems turn upon the unsystematic way the authors talk about the “self.” Throughout the article, the meaning of “self” oscillates between three specific biological understandings defined by the authors and far less precise usage (e.g., in claims like “interactions with microorganisms define the individual human self,” “profound implications…for our philosophical comprehension of the human self,” or in the essay’s title, “How the microbiome challenges our concept of self”). The context in which these looser attributions of “self” are deployed suggests at least three additional understandings that are more commonly the subject of humanistic or psychological inquiry. By conflating these various interpretations of self, Rees and colleagues create the impression that the microbiome’s impact on one specific biological notion automatically entails a similar impact on all notions of self (or perhaps on some unified notion). But these entailments are neither obvious nor argued for.

## Biological concepts of self

Rees and colleagues identify three biological bases of self—our immune systems, genetics, and brains—and argue that our microbiomes affect all three. The claimed effects range from our selves being “contingent on” our microbiomes to our microbiomes “co-constituting” ourselves. These claims concern the status of humans (and other multicellular creatures) as biological individuals. The nature of biological individuality has been extensively debated in biology as well as philosophy of biology (e.g., [2–3]). The key question about biological individuality raised by microbiome research is the extent to which our identity as an evolutionary or physiological individual depends on the microbes living in and on us.

One of the essay’s authors (Douglas) has effectively refuted claims that the microbiome and host form a single evolutionary individual [4]. The question of physiological unity is less clear. When the authors argue that our microbiomes “co-constitute” who we are, they most probably mean host and microbiome are physiologically and developmentally united, and that hosts such as humans would not function without their microbes. We agree that causal relationships between individual microbes or small groups of microbes and the host are very likely to impact how the host survives and thrives in daily life. But suggesting that the whole microbiome is causal is problematic (e.g., “the microbiome also plays a central role in the three processes that have traditionally been said to define the human self”). We simply don’t know what most of our microbes are doing, how much they contribute, and whether their effects are indispensable to human biology. This is not merely a matter of missing knowledge. Many microbiome participants are known empirically and theoretically to be transient, opportunistic, or even inactive (e.g., [5–7]). They are thus as much “human constituting” as many other organismal aspects of our environment, such as ticks or fleas.

## Humanistic concepts of self

Although the authors acknowledge that the human sciences have addressed multiple understandings of self, they make no attempt to identify them. We find at least three in their essay. At times, the authors have in mind the uniqueness of the human species (“what it means to be human,” that “distinctive human traits set us apart from all other animals”). At other times, they focus on the individual first-person experience of being a self (“self-awareness, personality traits, and emotional state,” “traits that we consider to define our sense of self”). At still other times, they seem concerned with agency or free will (“our resident microbes orchestrate the adaptive immune system [and] influence the brain”).

Asking what it means to be human in any of those three senses abstracts away from the specifics of our biological constitution. These questions focus instead on who we are as self-cognizing individuals, with our rich diversity of mental, emotional, and cultural resources. Questions about biological constitution are still relevant: the material bases of mind and consciousness are deep and interesting questions. But these connections are not straightforward. Consider the burgeoning work in cognitive science and philosophy on “the extended mind” [8–9]. On these accounts, a smartphone can constitute part of our mind, which suggests that (counter to Rees and colleagues’ claims about biological concepts of self) the internal composition of our bodies is not the main criterion for constituting at least one sense of humanistic or psychological selfhood.

Crucially, the various concepts at play here are not competing attempts to define some unitary concept of “the individual human self,” nor do claims about one concept of self automatically apply to another. Each concept plays importantly different roles that span the natural and social sciences and humanities. For instance, we might establish the “identity” of a criminal through genetic material left at the scene and then excuse the crime on the basis of the criminal’s state of mind during the crime (she was not “herself”).

## Connecting science, humanities, and the microbiome

In pointing out the various biological, humanistic, and psychological senses in which Rees and colleagues seem to be talking about “self,” we are not advocating a rigid divide between science and humanities. We are challenging the authors’ suggestion that the humanities and arts need to be fundamentally overhauled in light of microbiome research (their call for a “whole new configuration of research” resulting in “microbial humanities”). Throughout the history of science, Western society has gained numerous transformative insights about what constitutes our bodies and brains. While hugely important for both science and society, these insights have an impact on the humanities and arts by informing their subject matter and inspiring them to confront new topics in new ways—not by fundamentally reshaping how they are done.

Take, for instance, the revolutionary discoveries in the 19th century that contagious diseases are caused by specific microbes and that microorganisms are everywhere. These findings did not overhaul the humanities and arts, fuse them with microbiology, or otherwise “reconfigure” their research—nor should they have. These various disciplines carry on as before, in broad strokes, while drawing new inspiration from microbiology and engaging with it as subject matter. Consider, for example, powerful late-19th-century literature inspired by the omnipresence of microbes (such as H.G. Wells’ 1897 *War of the Worlds*) or recent microbiology-focused work in the philosophy of biology [10].

We wholeheartedly agree with Rees and colleagues that the humanities and the natural sciences should not operate in isolation from one another. Our endeavours in the humanities must keep pace with our best scientific understanding of the world, and some issues are best addressed by researchers from both sides joining forces. But one of the biggest barriers to productive interdisciplinary interactions is that terms have different meanings and connotations, and play different theoretical roles both within and across the sciences and humanities. Consider, for example, the varied uses of the term “gene” within biology [11] or the term “altruism” within evolutionary biology as opposed to psychology and moral philosophy [12]. To foster positive interactions between the humanities and sciences, an essential first step is to understand how terms such as “self” or “what it means to be human” are being used and what role they play in explanations and theories [13]. Without such an understanding, any claims about the microbiome’s consequences for these concepts are at best premature. We are currently unpersuaded that microbiome research has anything like the impact Rees and colleagues suggest it does on the six concepts of “self” identified above, nor that it justifies the disciplinary reconfiguration they call for.
